# Supplementary material for: Assessing Trunk Cross-Section Geometry and Spinal Postures with Noninvasive 3D Surface Topography: A Study of 108 Healthy Young Adults
Source: Sensors (Basel). 2025 Oct 28;25(21):6626. doi: 10.3390/s25216626 (PMC12608436; doi:10.3390/s25216626)
Supplement: Supplementary file 1 [file sensors-25-06626-s001.zip › sensors-3925069-supplementary.pdf]

Supplementary Material

for the manuscript:
"Assessing Trunk Cross-Section Geometry and Spinal Posture with Non-Invasive 3D Surface Topography: A Study in 108 Healthy Young Adults"

Table S1. Extended descriptive statistics

Normative TorsoScan values (N=108) with mean±SD, median, range, and percentiles (2.5th, 25th, 75th, 97.5th).

Variable	Mean	SD	Median	Min	Max	P2.5	P25	P75	P97.5
T1 Sagital	129.34	22.47	128.0	89.0	194.0	92.35	113.75	141.75	184.32
T1 Coronal	286.06	60.11	280.0	173.0	418.0	182.7	243.75	320.5	403.32
T1 Area	268.31	101.65	247.45	123.7	613.0	130.97	193.28	324.95	501.81
T4 Sagital	169.3	25.77	167.0	122.0	238.0	128.7	150.0	185.0	230.0
T4 Coronal	426.81	48.87	428.5	162.0	537.0	361.75	398.0	460.25	504.98
T4 Area	615.62	151.32	594.6	305.2	1130.5	396.64	495.45	701.68	942.05
T8 Sagital	216.78	32.64	212.5	157.0	336.0	167.68	196.0	232.5	295.28
T8 Coronal	372.92	54.99	363.5	258.0	532.0	284.82	331.75	412.25	473.32
T8 Area	748.58	188.5	720.15	404.1	1530.3	474.18	619.62	860.92	1255.68
T12	215.1	38.92	208.5	107.	370.0	157.7	191.0	235.2	313.95

Sagital	4			0		5		5	
T12 Coronal	310.74	45.34	308.5	223.0	459.0	241.68	279.25	339.5	404.75
T12 Area	580.95	172.1	545.5	274.6	1403.1	352.98	462.6	667.45	1002.14

Table S2. Full gender-stratified results with 95% CI

Expanded results by sex with mean±SD, mean difference (men − women), Cohen's d with 95% CI, two-sided Welch's t-test p-values, and FDR-corrected q-values (Benjamini–Hochberg).

Variable	Men mean ± SD	Women mean ± SD	Mean diff (M−F)	Cohen's d (95% CI)	p	q (FDR)
T1 Sagital	136.33 ± 22.62	124.16 ± 21.07	12.16	0.56 (0.17 to 0.95)	0.0055	0.0073
T1 Coronal	297.09 ± 59.02	277.89 ± 60.07	19.2	0.32 (-0.06 to 0.71)	0.1003	0.1003
T1 Area	293.01 ± 102.71	249.98 ± 97.68	43.03	0.43 (0.05 to 0.82)	0.0304	0.0339
T4 Sagital	175.61 ± 27.15	164.61 ± 23.85	11.0	0.43 (0.05 to 0.82)	0.0311	0.0339
T4 Coronal	451.20 ± 53.19	408.71 ± 36.35	42.49	0.96 (0.56 to 1.36)	1.302e-05	3.905e-05
T4 Area	697.39 ± 148.23	554.94 ± 123.16	142.45	1.06 (0.65 to 1.47)	8.776e-07	3.510e-06
T8 Sagital	229.37 ± 35.11	207.44 ± 27.41	21.93	0.71 (0.32 to 1.10)	7.143e-04	0.0011
T8 Coronal	409.13 ± 46.45	346.05 ± 44.61	63.08	1.39 (0.96 to 1.81)	2.296e-10	2.756e-09

T8 Area	864.28 ± 189.97	662.73 ± 134.61	201.55	1.26 (0.84 to 1.67)	3.331e-08	1.999e-07
T12 Sagital	231.22 ± 41.65	203.21 ± 32.21	28.01	0.77 (0.37 to 1.16)	2.809e-04	5.619e-04
T12 Coronal	327.96 ± 39.39	297.97 ± 45.53	29.99	0.70 (0.30 to 1.09)	3.998e-04	6.854e-04
T12 Area	664.98 ± 189.54	518.61 ± 127.19	146.38	0.93 (0.53 to 1.34)	2.180e-05	5.231e-05

Table S3. Pearson correlation matrix

All pairwise Pearson correlations between TorsoScan and DIERS Formetric parameters, showing correlation coefficient r, raw p-value, and FDR-adjusted q-value (Benjamini–Hochberg).

TorsoScan parameter	Formetric parameter	r	p	q (FDR)
T1 Sagittal diameter (mm)	Thoracic kyphosis angle VP–T12 [°]	0.3	0.0019	0.0107
T1 Sagittal diameter (mm)	Lumbar lordosis angle T12–DM [°]	0.04	0.6668	0.7090
T1 Sagittal diameter (mm)	Lateral deviation VP–DM (max) [mm]	-0.17	0.0839	0.1601
T1 Sagittal diameter (mm)	Surface rotation (max) [°]	0.06	0.5196	0.5743
T1 Sagittal diameter (mm)	Cervical arrow [mm]	0.3	0.0019	0.0107
T1 Sagittal	Pelvic obliquity (symmetry	-0.03	0.7623	0.8005

diameter (mm)	line) [°]			
T1 Sagittal diameter (mm)	Trunk length VP–DM [mm]	0.07	0.4706	0.5271
T1 Coronal diameter (mm)	Thoracic kyphosis angle VP–T12 [°]	0.08	0.4098	0.4781
T1 Coronal diameter (mm)	Lumbar lordosis angle T12–DM [°]	-0.09	0.3808	0.4546
T1 Coronal diameter (mm)	Lateral deviation VP–DM (max) [mm]	-0.08	0.4190	0.4822
T1 Coronal diameter (mm)	Surface rotation (max) [°]	0.22	0.0201	0.0643
T1 Coronal diameter (mm)	Cervical arrow [mm]	0.03	0.7906	0.8199
T1 Coronal diameter (mm)	Pelvic obliquity (symmetry line) [°]	-0.12	0.2152	0.2964
T1 Coronal diameter (mm)	Trunk length VP–DM [mm]	0.14	0.1413	0.2082
T1 Cross-sectional area (cm^2)	Thoracic kyphosis angle VP–T12 [°]	0.14	0.1599	0.2315
T1 Cross-sectional area (cm^2)	Lumbar lordosis angle T12–DM [°]	-0.04	0.6512	0.7013
T1 Cross-sectional area (cm^2)	Lateral deviation VP–DM (max) [mm]	-0.11	0.2362	0.3200
T1 Cross-sectional area (cm^2)	Surface rotation (max) [°]	0.15	0.1166	0.1886

T1 Cross-sectional area (cm^2)	Cervical arrow [mm]	0.11	0.2739	0.3551
T1 Cross-sectional area (cm^2)	Pelvic obliquity (symmetry line) [°]	-0.1	0.3017	0.3727
T1 Cross-sectional area (cm^2)	Trunk length VP–DM [mm]	0.1	0.2977	0.3727
T4 Sagittal diameter (mm)	Thoracic kyphosis angle VP–T12 [°]	0.16	0.1005	0.1759
T4 Sagittal diameter (mm)	Lumbar lordosis angle T12–DM [°]	-0.02	0.8347	0.8508
T4 Sagittal diameter (mm)	Lateral deviation VP–DM (max) [mm]	-0.17	0.0759	0.1487
T4 Sagittal diameter (mm)	Surface rotation (max) [°]	0.11	0.2790	0.3551
T4 Sagittal diameter (mm)	Cervical arrow [mm]	0.15	0.1305	0.1957
T4 Sagittal diameter (mm)	Pelvic obliquity (symmetry line) [°]	-0.11	0.2790	0.3551
T4 Sagittal diameter (mm)	Trunk length VP–DM [mm]	0.13	0.1831	0.2564
T4 Coronal diameter (mm)	Thoracic kyphosis angle VP–T12 [°]	-0.0	0.9629	0.9629
T4 Coronal diameter (mm)	Lumbar lordosis angle T12–DM [°]	-0.32	7.834e-04	0.0066

T4 Coronal diameter (mm)	Lateral deviation VP–DM (max) [mm]	-0.08	0.3843	0.4546
T4 Coronal diameter (mm)	Surface rotation (max) [°]	0.2	0.0411	0.1115
T4 Coronal diameter (mm)	Cervical arrow [mm]	0.24	0.0127	0.0485
T4 Coronal diameter (mm)	Pelvic obliquity (symmetry line) [°]	-0.26	0.0056	0.0248
T4 Coronal diameter (mm)	Trunk length VP–DM [mm]	0.41	1.259e-05	5.290e-04
T4 Cross-sectional area (cm^2)	Thoracic kyphosis angle VP–T12 [°]	0.05	0.5762	0.6286
T4 Cross-sectional area (cm^2)	Lumbar lordosis angle T12–DM [°]	-0.26	0.0060	0.0252
T4 Cross-sectional area (cm^2)	Lateral deviation VP–DM (max) [mm]	-0.15	0.1206	0.1911
T4 Cross-sectional area (cm^2)	Surface rotation (max) [°]	0.18	0.0560	0.1223
T4 Cross-sectional area (cm^2)	Cervical arrow [mm]	0.24	0.0116	0.0463
T4 Cross-sectional area (cm^2)	Pelvic obliquity (symmetry line) [°]	-0.29	0.0024	0.0121
T4 Cross-sectional area (cm^2)	Trunk length VP–DM [mm]	0.34	2.621e-04	0.0031

T8 Sagittal diameter (mm)	Thoracic kyphosis angle VP–T12 [°]	0.18	0.0604	0.1238
T8 Sagittal diameter (mm)	Lumbar lordosis angle T12–DM [°]	-0.11	0.2428	0.3237
T8 Sagittal diameter (mm)	Lateral deviation VP–DM (max) [mm]	-0.22	0.0205	0.0643
T8 Sagittal diameter (mm)	Surface rotation (max) [°]	0.16	0.1076	0.1844
T8 Sagittal diameter (mm)	Cervical arrow [mm]	0.3	0.0015	0.0098
T8 Sagittal diameter (mm)	Pelvic obliquity (symmetry line) [°]	-0.16	0.0914	0.1695
T8 Sagittal diameter (mm)	Trunk length VP–DM [mm]	0.19	0.0526	0.1223
T8 Coronal diameter (mm)	Thoracic kyphosis angle VP–T12 [°]	0.18	0.0558	0.1223
T8 Coronal diameter (mm)	Lumbar lordosis angle T12–DM [°]	-0.31	9.122e-04	0.0070
T8 Coronal diameter (mm)	Lateral deviation VP–DM (max) [mm]	-0.08	0.4295	0.4875
T8 Coronal diameter (mm)	Surface rotation (max) [°]	-0.02	0.8407	0.8508
T8 Coronal diameter (mm)	Cervical arrow [mm]	0.47	3.296e-07	2.769e-05
T8 Coronal	Pelvic obliquity (symmetry	-0.37	9.058e-05	0.0016

diameter (mm)	line) [°]			
T8 Coronal diameter (mm)	Trunk length VP–DM [mm]	0.23	0.0145	0.0509
T8 Cross-sectional area (cm^2)	Thoracic kyphosis angle VP–T12 [°]	0.15	0.1167	0.1886
T8 Cross-sectional area (cm^2)	Lumbar lordosis angle T12–DM [°]	-0.29	0.0023	0.0120
T8 Cross-sectional area (cm^2)	Lateral deviation VP–DM (max) [mm]	-0.21	0.0318	0.0891
T8 Cross-sectional area (cm^2)	Surface rotation (max) [°]	0.16	0.0928	0.1695
T8 Cross-sectional area (cm^2)	Cervical arrow [mm]	0.39	2.905e-05	8.133e-04
T8 Cross-sectional area (cm^2)	Pelvic obliquity (symmetry line) [°]	-0.33	4.735e-04	0.0044
T8 Cross-sectional area (cm^2)	Trunk length VP–DM [mm]	0.31	0.0013	0.0092
T12 Sagittal diameter (mm)	Thoracic kyphosis angle VP–T12 [°]	0.15	0.1260	0.1924
T12 Sagittal diameter (mm)	Lumbar lordosis angle T12–DM [°]	-0.15	0.1155	0.1886
T12 Sagittal diameter (mm)	Lateral deviation VP–DM (max) [mm]	-0.22	0.0207	0.0643
T12 Sagittal	Surface rotation (max)	0.13	0.1775	0.2528

diameter (mm)	[°]			
T12 Sagittal diameter (mm)	Cervical arrow [mm]	0.33	4.668e-04	0.0044
T12 Sagittal diameter (mm)	Pelvic obliquity (symmetry line) [°]	-0.15	0.1245	0.1924
T12 Sagittal diameter (mm)	Trunk length VP–DM [mm]	0.18	0.0553	0.1223
T12 Coronal diameter (mm)	Thoracic kyphosis angle VP–T12 [°]	0.24	0.0142	0.0509
T12 Coronal diameter (mm)	Lumbar lordosis angle T12–DM [°]	-0.18	0.0589	0.1236
T12 Coronal diameter (mm)	Lateral deviation VP–DM (max) [mm]	-0.21	0.0287	0.0841
T12 Coronal diameter (mm)	Surface rotation (max) [°]	0.16	0.0959	0.1714
T12 Coronal diameter (mm)	Cervical arrow [mm]	0.36	1.217e-04	0.0017
T12 Coronal diameter (mm)	Pelvic obliquity (symmetry line) [°]	-0.19	0.0442	0.1160
T12 Coronal diameter (mm)	Trunk length VP–DM [mm]	0.09	0.3494	0.4254
T12 Cross-sectional area (cm^2)	Thoracic kyphosis angle VP–T12 [°]	0.21	0.0290	0.0841
T12 Cross-sectional area (cm^2)	Lumbar lordosis angle T12–DM [°]	-0.19	0.0517	0.1223

T12 Cross-sectional area (cm^2)	Lateral deviation VP–DM (max) [mm]	-0.27	0.0043	0.0199
T12 Cross-sectional area (cm^2)	Surface rotation (max) [°]	0.17	0.0761	0.1487
T12 Cross-sectional area (cm^2)	Cervical arrow [mm]	0.37	9.411e-05	0.0016
T12 Cross-sectional area (cm^2)	Pelvic obliquity (symmetry line) [°]	-0.19	0.0507	0.1223
T12 Cross-sectional area (cm^2)	Trunk length VP–DM [mm]	0.18	0.0568	0.1223

Table S4. Partial correlation matrix (sex-adjusted)

Partial correlations between TorsoScan and DIERS Formetric parameters controlling for sex. Values shown are Pearson partial correlation coefficient (r), raw p-value, and FDR-adjusted q-value (Benjamini–Hochberg).

TorsoScan	Formetric	r	p	q (FDR)
T1 Sagittal diameter (mm)	Thoracic kyphosis angle VP–T12 [°]	0.31	0.0013	0.1068
T1 Sagittal diameter (mm)	Lumbar lordosis angle T12–DM [°]	0.18	0.0579	0.2116
T1 Sagittal diameter (mm)	Lateral deviation VP–DM (max) [mm]	-0.15	0.1242	0.3162
T1 Sagittal diameter (mm)	Surface rotation (max)	0.06	0.5249	0.8008

	[°]			
T1 Sagittal diameter (mm)	Cervical arrow [mm]	0.2	0.0407	0.2116
T1 Sagittal diameter (mm)	Pelvic obliquity (symmetry line) [°]	0.12	0.2301	0.4955
T1 Sagittal diameter (mm)	Trunk length VP–DM [mm]	-0.09	0.3785	0.6832
T1 Coronal diameter (mm)	Thoracic kyphosis angle VP–T12 [°]	0.08	0.4055	0.6952
T1 Coronal diameter (mm)	Lumbar lordosis angle T12–DM [°]	-0.02	0.8515	0.9754
T1 Coronal diameter (mm)	Lateral deviation VP–DM (max) [mm]	-0.07	0.5021	0.7810
T1 Coronal diameter (mm)	Surface rotation (max) [°]	0.22	0.0195	0.1883
T1 Coronal diameter (mm)	Cervical arrow [mm]	-0.06	0.5540	0.8165
T1 Coronal diameter (mm)	Pelvic obliquity (symmetry line) [°]	-0.05	0.5970	0.8304
T1 Coronal diameter (mm)	Trunk length VP–DM [mm]	0.07	0.4666	0.7395
T1 Cross-sectional area (cm^2)	Thoracic kyphosis angle VP–T12 [°]	0.14	0.1517	0.3747
T1 Cross-sectional area (cm^2)	Lumbar lordosis angle T12–DM [°]	0.05	0.5801	0.8304

T1 Cross-sectional area (cm^2)	Lateral deviation VP–DM (max) [mm]	-0.1	0.3105	0.6065
T1 Cross-sectional area (cm^2)	Surface rotation (max) [°]	0.15	0.1141	0.2995
T1 Cross-sectional area (cm^2)	Cervical arrow [mm]	0.01	0.9448	0.9941
T1 Cross-sectional area (cm^2)	Pelvic obliquity (symmetry line) [°]	-0.0	0.9984	0.9984
T1 Cross-sectional area (cm^2)	Trunk length VP–DM [mm]	-0.01	0.9147	0.9941
T4 Sagittal diameter (mm)	Thoracic kyphosis angle VP–T12 [°]	0.16	0.0936	0.2782
T4 Sagittal diameter (mm)	Lumbar lordosis angle T12–DM [°]	0.08	0.4014	0.6952
T4 Sagittal diameter (mm)	Lateral deviation VP–DM (max) [mm]	-0.16	0.1057	0.2865
T4 Sagittal diameter (mm)	Surface rotation (max) [°]	0.11	0.2791	0.5787
T4 Sagittal diameter (mm)	Cervical arrow [mm]	0.05	0.5883	0.8304
T4 Sagittal diameter (mm)	Pelvic obliquity (symmetry line) [°]	-0.0	0.9602	0.9941
T4 Sagittal diameter (mm)	Trunk length VP–DM [mm]	0.02	0.8208	0.9576

T4 Coronal diameter (mm)	Thoracic kyphosis angle VP–T12 [°]	-0.01	0.9514	0.9941
T4 Coronal diameter (mm)	Lumbar lordosis angle T12–DM [°]	-0.16	0.0960	0.2782
T4 Coronal diameter (mm)	Lateral deviation VP–DM (max) [mm]	-0.05	0.6030	0.8304
T4 Coronal diameter (mm)	Surface rotation (max) [°]	0.21	0.0270	0.2116
T4 Coronal diameter (mm)	Cervical arrow [mm]	0.04	0.6703	0.8663
T4 Coronal diameter (mm)	Pelvic obliquity (symmetry line) [°]	-0.07	0.4426	0.7150
T4 Coronal diameter (mm)	Trunk length VP–DM [mm]	0.24	0.0142	0.1883
T4 Cross-sectional area (cm^2)	Thoracic kyphosis angle VP–T12 [°]	0.06	0.5339	0.8008
T4 Cross-sectional area (cm^2)	Lumbar lordosis angle T12–DM [°]	-0.07	0.4416	0.7150
T4 Cross-sectional area (cm^2)	Lateral deviation VP–DM (max) [mm]	-0.12	0.2062	0.4557
T4 Cross-sectional area (cm^2)	Surface rotation (max) [°]	0.2	0.0355	0.2116
T4 Cross-sectional area (cm^2)	Cervical arrow [mm]	0.02	0.8033	0.9504

T4 Cross-sectional area (cm^2)	Pelvic obliquity (symmetry line) [°]	-0.08	0.3823	0.6832
T4 Cross-sectional area (cm^2)	Trunk length VP–DM [mm]	0.13	0.1702	0.3864
T8 Sagittal diameter (mm)	Thoracic kyphosis angle VP–T12 [°]	0.19	0.0469	0.2116
T8 Sagittal diameter (mm)	Lumbar lordosis angle T12–DM [°]	0.04	0.7011	0.8789
T8 Sagittal diameter (mm)	Lateral deviation VP–DM (max) [mm]	-0.21	0.0332	0.2116
T8 Sagittal diameter (mm)	Surface rotation (max) [°]	0.16	0.0957	0.2782
T8 Sagittal diameter (mm)	Cervical arrow [mm]	0.17	0.0758	0.2546
T8 Sagittal diameter (mm)	Pelvic obliquity (symmetry line) [°]	-0.01	0.9576	0.9941
T8 Sagittal diameter (mm)	Trunk length VP–DM [mm]	0.02	0.8709	0.9754
T8 Coronal diameter (mm)	Thoracic kyphosis angle VP–T12 [°]	0.22	0.0202	0.1883
T8 Coronal diameter (mm)	Lumbar lordosis angle T12–DM [°]	-0.09	0.3516	0.6624
T8 Coronal diameter (mm)	Lateral deviation VP–DM (max) [mm]	-0.03	0.7516	0.9150

T8 Coronal diameter (mm)	Surface rotation (max) [°]	-0.03	0.7442	0.9150
T8 Coronal diameter (mm)	Cervical arrow [mm]	0.27	0.0045	0.1704
T8 Coronal diameter (mm)	Pelvic obliquity (symmetry line) [°]	-0.13	0.1690	0.3864
T8 Coronal diameter (mm)	Trunk length VP–DM [mm]	-0.09	0.3548	0.6624
T8 Cross-sectional area (cm^2)	Thoracic kyphosis angle VP–T12 [°]	0.18	0.0653	0.2285
T8 Cross-sectional area (cm^2)	Lumbar lordosis angle T12–DM [°]	-0.08	0.4215	0.7082
T8 Cross-sectional area (cm^2)	Lateral deviation VP–DM (max) [mm]	-0.19	0.0513	0.2116
T8 Cross-sectional area (cm^2)	Surface rotation (max) [°]	0.18	0.0557	0.2116
T8 Cross-sectional area (cm^2)	Cervical arrow [mm]	0.18	0.0564	0.2116
T8 Cross-sectional area (cm^2)	Pelvic obliquity (symmetry line) [°]	-0.1	0.2825	0.5787
T8 Cross-sectional area (cm^2)	Trunk length VP–DM [mm]	0.04	0.6928	0.8789
T12 Sagittal diameter (mm)	Thoracic kyphosis angle VP–T12 [°]	0.16	0.1027	0.2865

T12 Sagittal diameter (mm)	Lumbar lordosis angle T12–DM [°]	0.0	0.9704	0.9941
T12 Sagittal diameter (mm)	Lateral deviation VP–DM (max) [mm]	-0.2	0.0339	0.2116
T12 Sagittal diameter (mm)	Surface rotation (max) [°]	0.14	0.1618	0.3864
T12 Sagittal diameter (mm)	Cervical arrow [mm]	0.2	0.0428	0.2116
T12 Sagittal diameter (mm)	Pelvic obliquity (symmetry line) [°]	0.03	0.7880	0.9456
T12 Sagittal diameter (mm)	Trunk length VP–DM [mm]	-0.0	0.9824	0.9943
T12 Coronal diameter (mm)	Thoracic kyphosis angle VP–T12 [°]	0.25	0.0095	0.1704
T12 Coronal diameter (mm)	Lumbar lordosis angle T12–DM [°]	-0.05	0.6323	0.8424
T12 Coronal diameter (mm)	Lateral deviation VP–DM (max) [mm]	-0.19	0.0462	0.2116
T12 Coronal diameter (mm)	Surface rotation (max) [°]	0.17	0.0850	0.2745
T12 Coronal diameter (mm)	Cervical arrow [mm]	0.25	0.0101	0.1704
T12 Coronal diameter (mm)	Pelvic obliquity (symmetry line) [°]	-0.05	0.6419	0.8424
T12 Coronal	Trunk length	-0.1	0.3024	0.6049

diameter (mm)	VP–DM [mm]			
T12 Cross-sectional area (cm^2)	Thoracic kyphosis angle VP–T12 [°]	0.23	0.0162	0.1883
T12 Cross-sectional area (cm^2)	Lumbar lordosis angle T12–DM [°]	-0.01	0.9587	0.9941
T12 Cross-sectional area (cm^2)	Lateral deviation VP–DM (max) [mm]	-0.26	0.0066	0.1704
T12 Cross-sectional area (cm^2)	Surface rotation (max) [°]	0.18	0.0569	0.2116
T12 Cross-sectional area (cm^2)	Cervical arrow [mm]	0.21	0.0313	0.2116
T12 Cross-sectional area (cm^2)	Pelvic obliquity (symmetry line) [°]	0.02	0.8706	0.9754
T12 Cross-sectional area (cm^2)	Trunk length VP–DM [mm]	-0.05	0.6250	0.8424

Table S5. Sex-stratified correlation comparisons

Correlation strength comparisons between men and women for all TorsoScan–DIERS parameter pairs. Values shown are Pearson correlation coefficients (r) for men and women, Fisher r-to-z test statistic (z), two-sided p-value, and FDR-adjusted q-value (Benjamini–Hochberg).

TorsoScan	Formetric	r (Men)	r (Women)	z	p	q (FDR)
T1 Sagittal diameter (mm)	Thoracic kyphosis angle VP–	0.33	0.29	0.23	0.8210	0.9854

	T12 [°]					
T1 Sagittal diameter (mm)	Lumbar lordosis angle T12–DM [°]	0.2	0.17	0.15	0.8841	0.9854
T1 Sagittal diameter (mm)	Lateral deviation VP–DM (max) [mm]	-0.11	-0.18	0.32	0.7455	0.9854
T1 Sagittal diameter (mm)	Surface rotation (max) [°]	0.03	0.08	-0.28	0.7805	0.9854
T1 Sagittal diameter (mm)	Cervical arrow [mm]	0.08	0.3	-1.11	0.2654	0.9854
T1 Sagittal diameter (mm)	Pelvic obliquity (symmetry line) [°]	0.12	0.11	0.05	0.9589	0.9854
T1 Sagittal diameter (mm)	Trunk length VP–DM [mm]	-0.15	-0.03	-0.58	0.5587	0.9854
T1 Coronal diameter (mm)	Thoracic kyphosis angle VP–T12 [°]	0.2	0.0	0.99	0.3198	0.9854
T1 Coronal diameter (mm)	Lumbar lordosis angle T12–DM [°]	0.12	-0.1	1.1	0.2702	0.9854
T1 Coronal diameter (mm)	Lateral deviation VP–DM (max) [mm]	-0.1	-0.04	-0.27	0.7900	0.9854

T1 Coronal diameter (mm)	Surface rotation (max) [°]	0.11	0.29	-0.95	0.3445	0.9854
T1 Coronal diameter (mm)	Cervical arrow [mm]	-0.1	-0.03	-0.34	0.7306	0.9854
T1 Coronal diameter (mm)	Pelvic obliquity (symmetry line) [°]	0.07	-0.12	0.98	0.3264	0.9854
T1 Coronal diameter (mm)	Trunk length VP–DM [mm]	-0.04	0.15	-0.97	0.3318	0.9854
T1 Cross-sectional area (cm^2)	Thoracic kyphosis angle VP–T12 [°]	0.21	0.09	0.58	0.5591	0.9854
T1 Cross-sectional area (cm^2)	Lumbar lordosis angle T12–DM [°]	0.15	-0.01	0.84	0.4010	0.9854
T1 Cross-sectional area (cm^2)	Lateral deviation VP–DM (max) [mm]	-0.06	-0.13	0.33	0.7415	0.9854
T1 Cross-sectional area (cm^2)	Surface rotation (max) [°]	0.06	0.21	-0.74	0.4593	0.9854
T1 Cross-sectional area (cm^2)	Cervical arrow [mm]	-0.1	0.1	-0.96	0.3351	0.9854
T1 Cross-sectional	Pelvic obliquity	0.11	-0.07	0.86	0.3876	0.9854

area (cm^2)	(symmetry line) [°]					
T1 Cross-sectional area (cm^2)	Trunk length VP–DM [mm]	-0.08	0.05	-0.64	0.5219	0.9854
T4 Sagittal diameter (mm)	Thoracic kyphosis angle VP–T12 [°]	0.16	0.17	-0.03	0.9761	0.9854
T4 Sagittal diameter (mm)	Lumbar lordosis angle T12–DM [°]	0.11	0.06	0.27	0.7846	0.9854
T4 Sagittal diameter (mm)	Lateral deviation VP–DM (max) [mm]	-0.12	-0.19	0.36	0.7166	0.9854
T4 Sagittal diameter (mm)	Surface rotation (max) [°]	0.13	0.09	0.18	0.8539	0.9854
T4 Sagittal diameter (mm)	Cervical arrow [mm]	-0.09	0.19	-1.44	0.1488	0.9854
T4 Sagittal diameter (mm)	Pelvic obliquity (symmetry line) [°]	0.03	-0.03	0.29	0.7748	0.9854
T4 Sagittal diameter (mm)	Trunk length VP–DM [mm]	-0.01	0.05	-0.3	0.7659	0.9854
T4 Coronal diameter (mm)	Thoracic kyphosis angle VP–T12 [°]	0.01	-0.03	0.2	0.8443	0.9854

T4 Coronal diameter (mm)	Lumbar lordosis angle T12–DM [°]	-0.09	-0.24	0.81	0.4156	0.9854
T4 Coronal diameter (mm)	Lateral deviation VP–DM (max) [mm]	-0.12	0.02	-0.69	0.4894	0.9854
T4 Coronal diameter (mm)	Surface rotation (max) [°]	0.14	0.29	-0.77	0.4391	0.9854
T4 Coronal diameter (mm)	Cervical arrow [mm]	-0.05	0.15	-1.0	0.3157	0.9854
T4 Coronal diameter (mm)	Pelvic obliquity (symmetry line) [°]	-0.01	-0.14	0.64	0.5212	0.9854
T4 Coronal diameter (mm)	Trunk length VP–DM [mm]	0.13	0.36	-1.22	0.2219	0.9854
T4 Cross-sectional area (cm^2)	Thoracic kyphosis angle VP–T12 [°]	0.1	0.03	0.38	0.7070	0.9854
T4 Cross-sectional area (cm^2)	Lumbar lordosis angle T12–DM [°]	-0.01	-0.13	0.61	0.5446	0.9854
T4 Cross-sectional area (cm^2)	Lateral deviation VP–DM (max)	-0.09	-0.15	0.33	0.7422	0.9854

	[mm]					
T4 Cross-sectional area (cm^2)	Surface rotation (max) [°]	0.13	0.26	-0.67	0.5023	0.9854
T4 Cross-sectional area (cm^2)	Cervical arrow [mm]	-0.07	0.12	-0.94	0.3464	0.9854
T4 Cross-sectional area (cm^2)	Pelvic obliquity (symmetry line) [°]	-0.0	-0.15	0.74	0.4565	0.9854
T4 Cross-sectional area (cm^2)	Trunk length VP–DM [mm]	0.09	0.18	-0.44	0.6588	0.9854
T8 Sagittal diameter (mm)	Thoracic kyphosis angle VP–T12 [°]	0.2	0.19	0.07	0.9431	0.9854
T8 Sagittal diameter (mm)	Lumbar lordosis angle T12–DM [°]	0.06	0.02	0.22	0.8242	0.9854
T8 Sagittal diameter (mm)	Lateral deviation VP–DM (max) [mm]	-0.22	-0.19	-0.17	0.8618	0.9854
T8 Sagittal diameter (mm)	Surface rotation (max) [°]	0.27	0.09	0.91	0.3607	0.9854
T8 Sagittal diameter (mm)	Cervical arrow [mm]	0.06	0.3	-1.25	0.2125	0.9854
T8 Sagittal diameter	Pelvic obliquity (symmetry	0.0	-0.01	0.06	0.9493	0.9854

(mm)	line) [°]					
T8 Sagittal diameter (mm)	Trunk length VP–DM [mm]	0.07	-0.04	0.56	0.5762	0.9854
T8 Coronal diameter (mm)	Thoracic kyphosis angle VP–T12 [°]	0.33	0.15	0.97	0.3329	0.9854
T8 Coronal diameter (mm)	Lumbar lordosis angle T12–DM [°]	-0.05	-0.12	0.35	0.7277	0.9854
T8 Coronal diameter (mm)	Lateral deviation VP–DM (max) [mm]	0.05	-0.09	0.7	0.4865	0.9854
T8 Coronal diameter (mm)	Surface rotation (max) [°]	0.03	-0.07	0.47	0.6377	0.9854
T8 Coronal diameter (mm)	Cervical arrow [mm]	0.22	0.32	-0.52	0.6042	0.9854
T8 Coronal diameter (mm)	Pelvic obliquity (symmetry line) [°]	-0.21	-0.09	-0.63	0.5290	0.9854
T8 Coronal diameter (mm)	Trunk length VP–DM [mm]	0.01	-0.17	0.86	0.3883	0.9854
T8 Cross-sectional area (cm^2)	Thoracic kyphosis angle VP–	0.23	0.14	0.48	0.6347	0.9854

	T12 [°]					
T8 Cross-sectional area (cm^2)	Lumbar lordosis angle T12–DM [°]	-0.06	-0.1	0.21	0.8315	0.9854
T8 Cross-sectional area (cm^2)	Lateral deviation VP–DM (max) [mm]	-0.12	-0.27	0.8	0.4235	0.9854
T8 Cross-sectional area (cm^2)	Surface rotation (max) [°]	0.2	0.19	0.06	0.9537	0.9854
T8 Cross-sectional area (cm^2)	Cervical arrow [mm]	0.15	0.23	-0.39	0.6932	0.9854
T8 Cross-sectional area (cm^2)	Pelvic obliquity (symmetry line) [°]	-0.09	-0.13	0.21	0.8325	0.9854
T8 Cross-sectional area (cm^2)	Trunk length VP–DM [mm]	0.11	-0.04	0.78	0.4378	0.9854
T12 Sagittal diameter (mm)	Thoracic kyphosis angle VP–T12 [°]	0.14	0.18	-0.23	0.8201	0.9854
T12 Sagittal diameter (mm)	Lumbar lordosis angle T12–DM [°]	-0.0	0.01	-0.07	0.9408	0.9854
T12 Sagittal diameter (mm)	Lateral deviation VP–DM (max) [mm]	-0.3	-0.11	-0.95	0.3414	0.9854

T12 Sagittal diameter (mm)	Surface rotation (max) [°]	0.2	0.09	0.54	0.5910	0.9854
T12 Sagittal diameter (mm)	Cervical arrow [mm]	0.08	0.31	-1.2	0.2300	0.9854
T12 Sagittal diameter (mm)	Pelvic obliquity (symmetry line) [°]	0.02	0.03	-0.02	0.9854	0.9854
T12 Sagittal diameter (mm)	Trunk length VP–DM [mm]	0.01	-0.01	0.11	0.9089	0.9854
T12 Coronal diameter (mm)	Thoracic kyphosis angle VP–T12 [°]	0.35	0.19	0.86	0.3906	0.9854
T12 Coronal diameter (mm)	Lumbar lordosis angle T12–DM [°]	0.04	-0.09	0.66	0.5107	0.9854
T12 Coronal diameter (mm)	Lateral deviation VP–DM (max) [mm]	-0.28	-0.13	-0.76	0.4465	0.9854
T12 Coronal diameter (mm)	Surface rotation (max) [°]	0.15	0.18	-0.14	0.8905	0.9854
T12 Coronal diameter (mm)	Cervical arrow [mm]	0.25	0.25	0.02	0.9850	0.9854

T12 Coronal diameter (mm)	Pelvic obliquity (symmetry line) [°]	0.01	-0.07	0.39	0.7000	0.9854
T12 Coronal diameter (mm)	Trunk length VP–DM [mm]	-0.03	-0.15	0.63	0.5280	0.9854
T12 Cross-sectional area (cm^2)	Thoracic kyphosis angle VP–T12 [°]	0.23	0.25	-0.12	0.9013	0.9854
T12 Cross-sectional area (cm^2)	Lumbar lordosis angle T12–DM [°]	-0.0	-0.01	0.03	0.9736	0.9854
T12 Cross-sectional area (cm^2)	Lateral deviation VP–DM (max) [mm]	-0.26	-0.27	0.05	0.9576	0.9854
T12 Cross-sectional area (cm^2)	Surface rotation (max) [°]	0.23	0.16	0.37	0.7142	0.9854
T12 Cross-sectional area (cm^2)	Cervical arrow [mm]	0.14	0.3	-0.83	0.4040	0.9854
T12 Cross-sectional area (cm^2)	Pelvic obliquity (symmetry line) [°]	0.02	0.01	0.04	0.9671	0.9854
T12 Cross-sectional area (cm^2)	Trunk length VP–DM [mm]	0.01	-0.11	0.6	0.5481	0.9854

Table S6. Full regression models

Regression models for all TorsoScan outcomes (cross-validated 10×5). Results are reported as β coefficients (clinical units) with 95% confidence intervals, together with model fit statistics R^2, RMSE, and MAE. All models controlled for sex. Predictors were selected using Lasso/Elastic Net, with final estimates from OLS.

Outcome (TorsoScan)	N	R^2 (10×5)	RMSE	MAE	Significant predictors (β, 95% CI, p)
T8 Coronal (mm)	108	0.185	44.9	36.0	Sex (+50.8 mm, p<0.01); Pelvic obliquity (−2.31 mm/°, p<0.05); Lumbar lordosis T12–DM (+4.46 mm/°, p<0.05)
T8 Area (cm^2)	108	0.068	154.7	119.7	Sex (+164.4 cm^2, p<0.01); T8 flexion/extension (+48.2, p<0.05); Flèche cervicale (+7.0 mm, p<0.05); T7 flexion/extension (−45.0, p<0.05)
T12 Coronal (mm)	108	0.068	40.1	32.5	Flèche cervicale (+1.0 mm, p<0.05); T11 rotation (+3.7 mm, p<0.05)
T12 Area (cm^2)	108	0.059	138.0	108.8	Sex (+83.8 cm^2, p<0.05); T9 flexion/extension (+93.4, p<0.05); T8 flexion/extension (−70.9, p<0.05); Flèche cervicale (+3.2 mm, p<0.05); T11

					flexion/extension (–24.3, $p<0.05$); T11 rotation (+13.8 mm, $p<0.05$)
T4 Area (cm^2)	108	0.008	131.2	104.0	Sex (+107.0 cm^2, $p<0.05$)
T4 Coronal (mm)	108	0.003	39.9	30.2	None significant

Figure S1. Distribution plots

Boxplots or violin plots for TorsoScan measures at Th1, Th4, Th8, Th12 by sex.

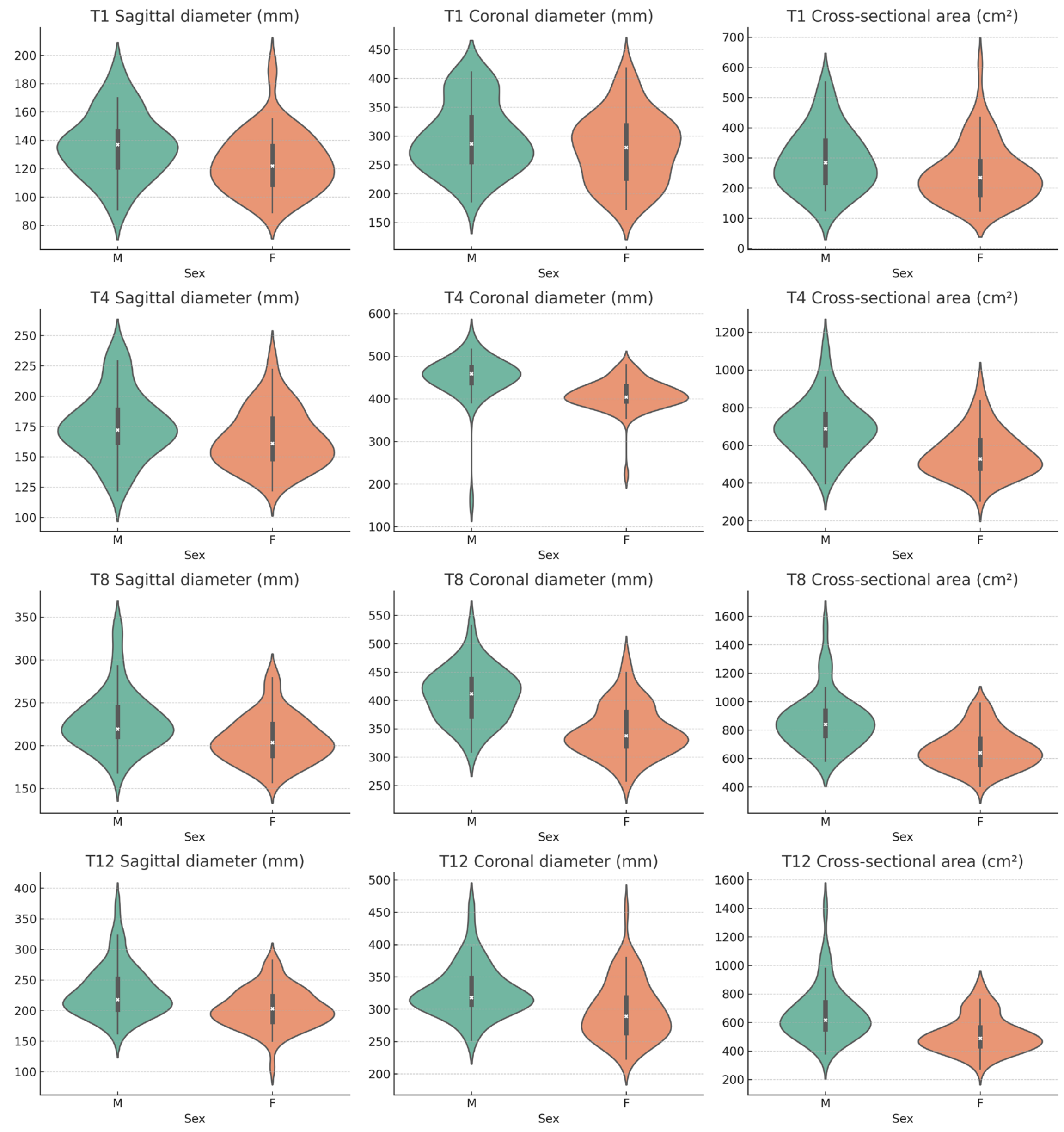

Violin plots with embedded boxplots showing the distribution of sagittal diameter, coronal diameter, and cross-sectional area at Th1, Th4, Th8, and Th12 levels in healthy young adults (N = 108). Male participants are shown in green and female participants in orange. Each violin represents the kernel density of the data; the central box indicates the interquartile range, the horizontal line inside the box marks the median, and the whiskers extend to 1.5× IQR. These plots illustrate the sex-specific differences in thoracic cross-sectional geometry, with men generally exhibiting larger coronal diameters and areas, especially at Th4–Th12, consistent with the results reported in Table 2.

Figure S2. Scatterplots for significant correlations

Scatterplots with regression lines and 95% CI for significant associations (e.g., Th4 coronal vs. lumbar lordosis).

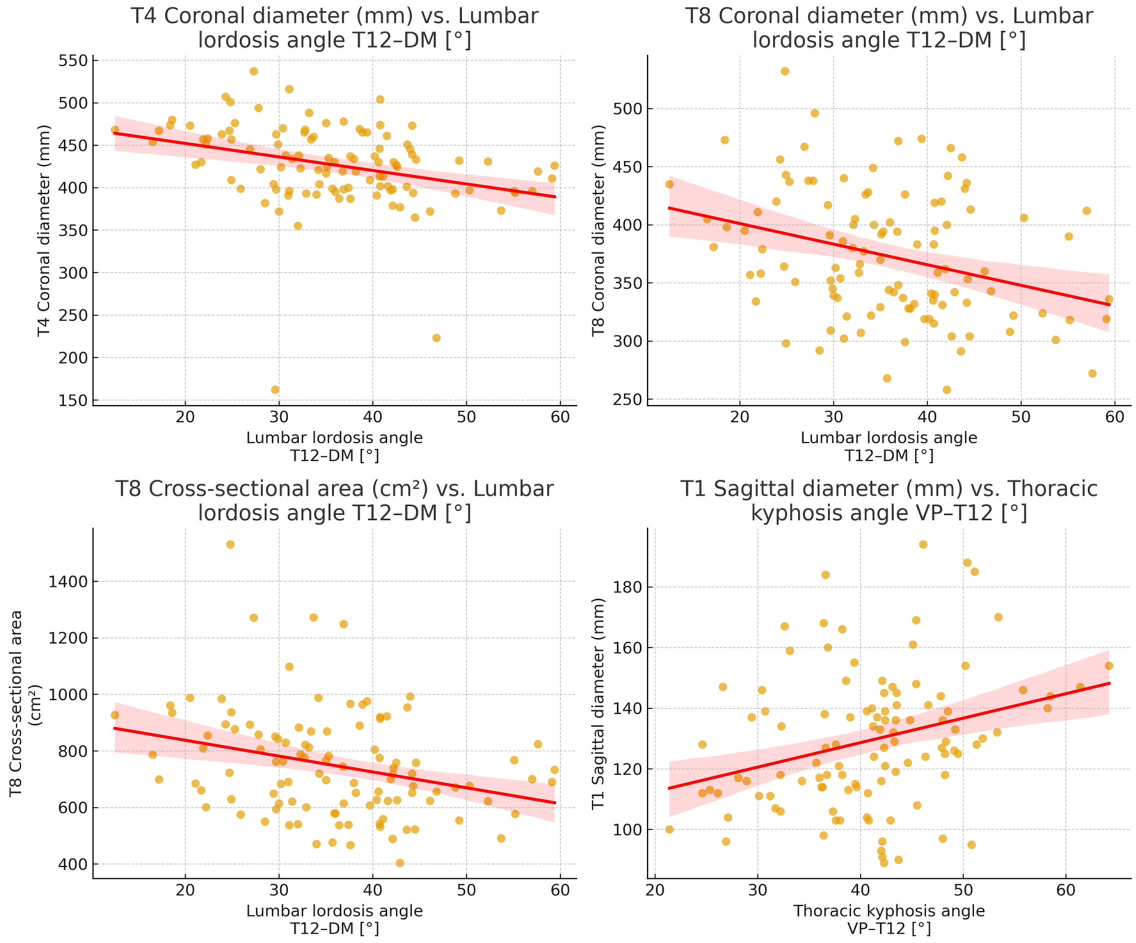

Scatterplots with linear regression lines (red) and 95% confidence intervals (shaded area) are shown for associations that remained significant after FDR correction: (a) T4 coronal diameter vs. lumbar lordosis angle T12–DM, (b) T8 coronal diameter vs. lumbar lordosis angle T12–DM, (c) T8 cross-sectional area vs. lumbar lordosis angle T12–DM, and (d) T1 sagittal diameter vs. thoracic kyphosis angle VP–T12. Each dot represents one participant (N = 108). Negative correlations with lumbar lordosis indicate narrower thoracic cross-sections in individuals with greater lumbar curvature, while the positive correlation with thoracic kyphosis reflects deeper anteroposterior thoracic dimensions.

Methodological Appendix

Cross-validation procedure

All regression models were evaluated using a repeated 10×5 cross-validation scheme. The full dataset (N = 108) was randomly partitioned into 10 folds; in each round, 9 folds were used for training and 1 for testing. This procedure was repeated across 5 iterations with different random splits to reduce variance due to fold assignment. Reported R^2, RMSE, and MAE are averaged across all test folds. This approach was chosen over a simple holdout to maximize stability of estimates given the moderate sample size.

Predictor selection

Independent variables were selected from DIERS Formetric parameters that showed at least moderate correlation with the TorsoScan outcome ($|r| \geq 0.30$).
Subsequently, Lasso and Elastic Net regularization were applied to reduce collinearity and overfitting. Optimal penalty parameters (λ, α) were determined via internal cross-validation. Predictors retained by the penalized regression were then entered into a final ordinary least squares (OLS) model, controlling for sex in all analyses. Regression coefficients are reported as β with 95% confidence intervals.

Multicollinearity diagnostics

Variance Inflation Factors (VIFs) were computed for all predictors in the final OLS models. No VIF exceeded the commonly used threshold of 5, indicating acceptable levels of collinearity. Predictors with excessively high VIFs during preliminary screening were excluded prior to final model estimation.

Homoscedasticity and residual diagnostics

Residuals were inspected visually (residual vs. fitted plots, Q–Q plots) and statistically tested. Breusch–Pagan tests did not indicate severe heteroscedasticity (all $p > 0.05$ after FDR correction). Normality of residuals was generally acceptable, though mild deviations were noted in models with low explanatory power (e.g., T12 area).

Software environment

All analyses were performed in Python 3.11 using the following packages:

- *pandas, numpy, scipy* (data processing and descriptive statistics)
- *statsmodels* (OLS regression, diagnostic tests, VIF)
- *scikit-learn* (cross-validation, Lasso, Elastic Net)
- *matplotlib, seaborn* (visualizations)
